# Results of intraocular lens implantation with capsular tension ring in subluxated crystalline or cataractous lenses in children

**DOI:** 10.4103/0301-4738.57149

**Published:** 2009

**Authors:** Pranab Das, Jagat Ram, Gagandeep Singh Brar, Mangat R Dogra

**Affiliations:** Department of Ophthalmology, Advanced Eye Centre, Post Graduate Institute of Medical Education and Research, Chandigarh-160 012, India

**Keywords:** Capsular tension ring, posterior capsular opacification, subluxated lens, traumatic cataract

## Abstract

**Purpose:**

To evaluate the outcome of intraocular lens (IOL) implantation using capsular tension ring (CTR) in subluxated crystalline or cataractous lenses in children.

**Setting:**

Tertiary care setting

**Materials and Methods:**

We prospectively studied 18 eyes of 15 children with subluxation of crystalline or cataractous lenses between 90° up to 210° after phacoemulsification, CTR and IOL implantation. Each child was examined for IOL centration, zonular dehiscence and posterior capsular opacification (PCO).

**Results:**

Age of the patient ranged between five to 15 years. Out of 18 eyes, seven had traumatic and 11 had spontaneous subluxation of crystalline or cataractous lens. Phacoemulsification was successfully performed with CTR implantation in the capsular bag. Intraoperative zonular dialysis occurred in two eyes. Anterior vitrectomy was performed in six eyes to manage vitreous prolapse. IOL implanted was polymethyl methacrylate (PMMA) in eight eyes, hydrophobic acrylic in seven and hydrophilic acrylic in three. Follow-up ranged from 24 months to 72 months. Sixteen eyes had a best corrected visual acuity of 20/40 or better. Nine eyes developed significant PCO and were managed with Neodymium Yttrium Aluminum Garnet (Nd:YAG) laser posterior capsulotomy. One eye with acrylic IOL in the capsular bag had IOL dislocation after two years which was managed with vitrectomy and secondary trans-scleral fixation of IOL.

**Conclusions:**

Phacoaspiration with CTR implantation makes capsular bag IOL fixation possible in most of the eyes with subluxated crystalline or cataractous lenses. PCO still remains a challenge in children with successful phacoaspiration with CTR implantation

Management of subluxated crystalline lens in children is a challenge. Surgical procedures such as intracapsular cataract extraction,[1] limbal or pars plana lensectomy and anterior vitrectomy[2] and suturing of the capsule to the sclera or the haptics to the sulcus[3] have been reported with several complications.[4] Recent reports of management of subluxated cataract by phacoemulsification with capsular tension ring (CTR) have shown encouraging results.[5,6]

Hara *et al*,[7] introduced the ring called “Equator ring” in 1991 for the first time for the maintenance of the completely circular contour of the capsular bag equator after cataract removal and to prevent posterior capsular opacification (PCO) by blocking the posterior movement of the lens epithelial cells at the equator. Nagamoto *et al*.[8] and Legler *et al*.[9] modified this ring to an open ring with a slim loop. The standard CTR is an open-ended, flexible horseshoe-shaped polymethyl methacrylate (PMMA) filament with two eyelets at their ends. CTR can be inserted into the capsular bag anytime after successful capsulorrhexis. Once positioned in the capsular bag, a CTR helps distribute the stress around the entire equatorial area. Because of this stress distribution, the likelihood of stretching or extension of zonular dialysis reduces significantly.[10] After surgery it maintains centration of intraocular lens (IOL) by keeping the capsular bag stretched. We evaluated the outcome of IOL implantation using CTR in subluxated clear or cataractous lenses in children.

## Materials and Methods

We prospectively studied children recruited between January 2001 to December 2004, aged five to 15 years with subluxation of crystalline or cataractous lens varying between 90° to 210° who underwent successful phacoemulsification with CTR and IOL implantation. This was a nonrandomized observational study conducted with institutional review board clearance. These were consecutive cases where a successful CTR and IOL implantation was achieved. Parents were informed about the available options which included phacoaspiration with an IOL and CTR implantation, or pars plana lensectomy and anterior vitrectomy with or without trans-scleral fixated IOL. Informed consent was obtained from parents before enrolling the child in the study after explaining the rationale of use of CTR, the nature of surgery, the associated risks, complications, and the expected visual outcome.

A complete ophthalmologic examination was done including best corrected visual acuity, applanation tonometry, slit-lamp biomicroscopy, presence or absence of vitreous in anterior chamber, and integrity of zonules. Degree of zonular dehiscence or separation was estimated using retro-illumination in slit-lamp biomicroscope after full pupillary dilatation. Detailed dilated fundus evaluation was done using + 90D lens or indirect ophthalmoscope. IOL power was calculated using SRK-II formula. In case of uncooperative children biometry was done under general anesthesia. B-scan ultrasound was done for posterior segment evaluation if the fundus was not visible. If subluxation was suspected to be due to any heritable disease, internist opinion was sought regarding the diagnosis of the disease.

All surgical procedures were performed under general anesthesia. Mydriasis was achieved by using a combination of tropicamide 0.8% and phenylephrine 5% and cyclopentolate 1% three times at intervals of half an hour before surgery.

A valvular incision was made superiorly preferably in the axis of intact zonule using 3.0 mm disposable keratome in all cases and two side port incisions were made. High-viscosity visco-elastic sodium hyaluronate 1.4% (Healon GV^®^, Advanced medical optics, Uppsala) was introduced into the anterior chamber through a side port incision. Continuous curvilinear capsulorrhexis (CCC) of 5 to 5.5 mm size was created with the help of a bent 26-gauge needle and Uttrata forceps. Flexible silicone iris hooks were used to stabilize the CCC margin in the area of the dehiscence in all cases. The stop on the hook was adjusted to hold the rhexis edge and stabilize the capsular bag. Subsequently a gentle multi-quadrant hydrodissection was performed. At this stage, the CTR (CTR-11, 13/11mm, Aurolab, Madurai, India) was introduced into the capsular bag with the help of Kelman-McPherson forceps and a second manipulating spatula to guide the device into the capsular bag. CTR of 13/11 mm size was used in all eyes. Subsequently, phacoemulsification was performed with low vacuum and low aspiration and low bottle height. The remaining cortical matter was removed by automated bimanual irrigation and aspiration. Vitreous in the anterior chamber at any stage of surgery was managed with a two-port anterior vitrectomy. The posterior capsule was left intact. In case of clear lens, the position of CTR was confirmed by retracting the iris with Kuglen's hook immediately after the implantation of the CTR and in cataractous lenses, it was done after aspiration of cortical matter. Posterior chamber (PC) IOL was implanted in the capsular bag placing the haptic in line with the area of zonular dehiscence. Depending upon the patient's affordability, all PMMA rigid IOL (Aurolab SQ3602, Madurai, Tamil Nadu, India), or hydrophobic acrylic IOL (Sensar Optiedge, AR40e, Advanced Medical Optic Santa Ana, California), or three-piece hydrophilic acrylic (RYCF, Care Group, Baroda India) was implanted. Residual visco-elastic was then removed. At the end of the procedure, centration and stability of the IOL was tested.

After surgery, patients were examined on slit-lamp biomicroscopy on Day 1, Day 7, Day 14, Day 28 and Day 90 and then six-monthly. At every visit the following parameters were noted: visual acuity, intraocular pressure, anterior chamber depth (symmetric/asymmetric), IOL centration, IOL tilt, capsular bag stability, zonular dehiscence and PCO. Patients' follow-up varied from 24 to 72 months.

## Results

Of the 15 children included in the study, there were 10 male and five female children. Age of the children ranged from five to 15 years with a mean age of 9.20 ± 3.25 years. Out of 18 eyes, seven had traumatic subluxated cataractous lens and 11 eyes had spontaneously subluxated crystalline or cataractous lens. Out of seven traumatic cataracts five were total cataract and two were posterior subcapsular cataract. The preoperative best corrected visual acuity (BCVA) ranged from HMCF (which is defined as perception of hand motion at two feet distance) to 20/80. The extent of dialysis ranged from 90° to 210°. Subluxation varied from 90° to 150° in nine eyes and more than 150° to 210° in nine eyes. In the area of zonular defect, zonules were totally absent in seven eyes and partially absent in five eyes and stretched in six eyes.

Preoperatively, vitreous was present in the anterior chamber in three eyes with traumatic subluxation of lens. None of the eyes with spontaneous subluxation had vitreous disturbances preoperatively.

Phacoaspiration with limbal incision was performed with CCC in all cases. In all eyes CTR was implanted in the capsular bag and no major complication was noted during CTR implantation. Intraoperative extension of dialysis occurred in two eyes, one eye had traumatic cataract with vitreous in anterior chamber at presentation with 150° of subluxation, and the other eye had a subluxated cataractous lens with 160° of zonular dialysis. In both the eyes zonular dialysis extended to 180° intraoperatively. Capsular bag collapse did not occur in any eye with CTR. No eye had posterior capsular tear. In six eyes anterior vitrectomy was performed to manage vitreous prolapse in the anterior chamber. Out of these six eyes, three eyes had vitreous in anterior chamber preoperatively and in the remaining three eyes vitreous herniation occurred intraoperatively. In all eyes IOL was implanted in the capsular bag with good centration. IOL implanted was rigid PMMA in eight eyes and acrylic foldable in 10 eyes (hydrophobic in seven and hydrophilic in three eyes).

Postoperatively minimal corneal edema, mainly stromal and near section, occurred in five eyes which resolved by first postoperative week. Three eyes with traumatic cataract had more than 2+ cells and flare at two weeks. In all cases anterior chamber inflammation subsided with topical steroids. Two eyes developed raised IOP due to residual visco-elastic in the anterior chamber and responded well to anti-glaucoma medications.

**Figure 1:**
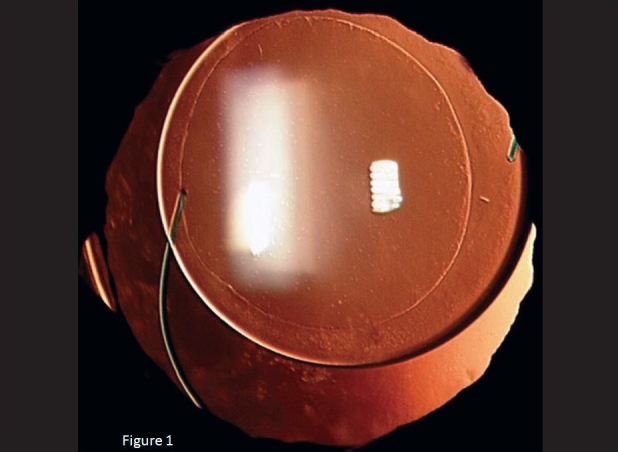
Well centered hydrophobic acrylic IOL implantation in the capsular bag in a child of 5years for approximately 150degree traumatic subluxation of crystalline lens. Continuous curvilinear capsulorhexis of approximately 5mm and also seen in the capsular bag is capsular tension ring

**Figure 2:**
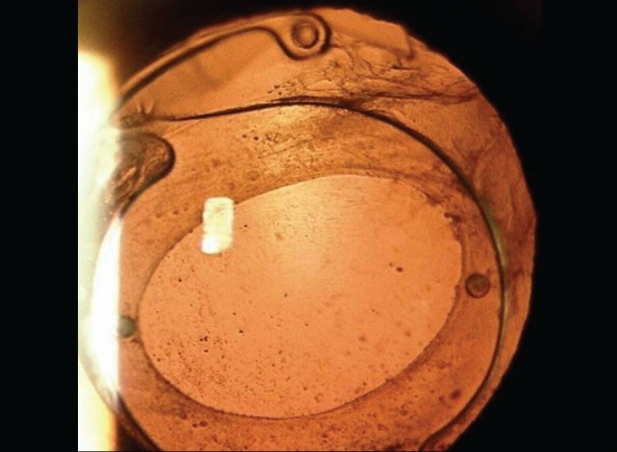
Well centred PMMA IOL in the capsular bag in 12year old child with traumatic subluxation of cataractous lens approximately 150degree. Note capsule tension ring superiorly

**Figure 3:**
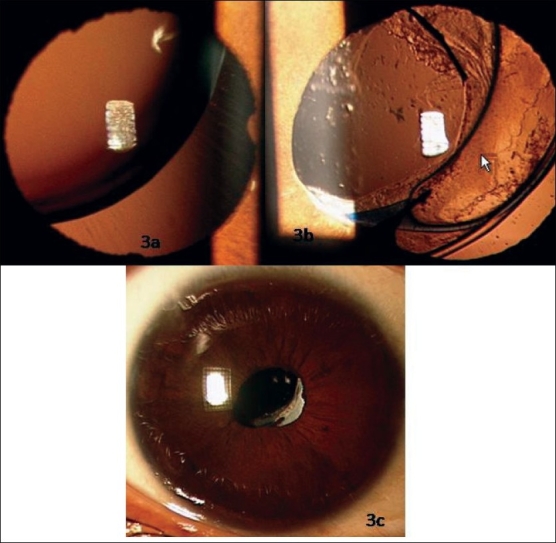
(a) Right eye of a 10 years old child with Marfan's syndrome with supero-temporal subluxation of crystalline lens of approximately 200degree. (b) Same eye as in figure 3a showing late decentration of IOL 5 years after implantation. Note clear visual axis after Nd: YAG laser capsulotomy for posterior capsular opacification. Capsular tension ring is also seen inferiorly. (c) Same eye with an undilated pupil, there was reasonable centration of IOL. Opacified anterior edge of the capsulorhexis is passing through the infero-nasal pupil which has helped to prevent diplopia

All the IOLs were well centered on the first postoperative day except for one case in which the IOL was decentered with edge of the IOL at the center of the pupil. On the third postoperative day, a second surgical procedure was done to re-centre the IOL. One haptic of the IOL was fixed to the sclera with 10-0 polypropylene monofilament suture. All the IOLs at last follow-up remained well centered [Figs. 1,2] except in an eye where there were increase in the decentration with increasing capsular bag fibrosis [Figs. 3].

Sixteen out of 18 (89%) eyes had a final BCVA of 20/40 or better at last follow-up. One eye that showed least improvement of vision (20/400) had a traumatic macular scar with choroidal tear involving the fovea which was not detected preoperatively because of cataractous lens and another eye with spontaneous subluxation of lens was amblyopic and had final visual acuity of 20/60.

Nine out of 18 eyes developed significant PCO. Three of the 10 eyes implanted with square edge hydrophobic/hydrophilic acrylic and six of the eight eyes implanted with round edge PMMA IOL developed visually significant PCO and were managed with Nd:YAG laser posterior capsulotomy. One eye of a child with 210° subluxation implanted with acrylic hydrophilic IOL dislocated with capsular bag into the vitreous cavity after four years. In this eye vitrectomy was done with removal of IOL with capsular bag and trans-scleral fixation of posterior chamber IOL.

## Discussion

Trauma is the most common cause of subluxation of crystalline lens. Common causes of spontaneous subluxation include Marfan's syndrome, Weill-Marchesani syndrome, and homocystinuria. In the present study, seven eyes (seven children) had subluxation due to trauma and in 11 eyes subluxation was spontaneous [Table 1]. Pars plana lensectomy with anterior vitrectomy had been employed for subluxated lens in children by various investigators with good visual outcome.[3,11] Although this procedure appears to give good visual outcomes, the patients remained aphakic postoperatively in all cases. The treatment of aphakia in children is a significant challenge. Spectacle correction is not suitable for unilateral aphakia especially after surgery for traumatic subluxation due to anisokenia. Contact lens wear has its own drawbacks including keratitis, corneal neovascularization, noncompliance, frequent lens change and cost. Surgical methods of aphakic correction are implantation of anterior chamber IOL and trans-scleral fixated IOL. Anterior chamber IOL fixation is not a preferred modality in the pediatric age group due to several postoperative complications like corneal decompensation, glaucoma and retinal detachments. Recently published studies reported late dislocation of IOL due to breakage of polypropylene sutures in scleral fixated posterior chamber IOL, especially in young patients.[12,13] Asadi *et al*, recently reported this disturbing late complication of trans-scleral fixated IOL in children. In their series late IOL dislocation due to breakage of polypropylene sutures was noted after seven to 10 years in six out of 25 eyes (24%).[14]

**Table 1:** Profile of children with subluxated cataract

Patient	Age	Sex	Diagnosis	Subluxation in degree	Laterality	Preop BCVA	Postop BCVA*	IOL	Complications	Follow-up (Mon)
1	14	M	Traumatic	90	UL†	HM	20/30	PMMA	PCO§	72
2	13	M	Traumatic	140	UL	CF	20/20	PMMA	-	51
3	9	F	Traumatic	120	UL	CF	20/30	PMMA	PCO	44
4	8	F	Idiopathic	150	UL	20/200	20/40	Acrylic	-	30
5	5	F	Traumatic	170	UL	HM	20/400	PMMA	-	45
6	12	M	Traumatic	150	UL	20/80	20/40	PMMA	PCO	33
7	7	F	Idiopathic	180	UL	20/60	20/40	Acrylic	-	42
8	5	M	Idiopathic	150	UL	20/200	20/60	PMMA	PCO	24
9	11	F	Idiopathic	140	UL	20/80	20/20	Acrylic	PCO	39
10	8	M	Traumatic	130	UL	20/200	20/20	Acrylic	PCO	36
11	5	M	Traumatic	150	UL	20/80	20/20	Acrylic	-	45
12	15	M	Idiopathic	180	UL	20/200	20/30	Acrylic	-	24
13	10	F	Marfan's	200 RE	‡BL	20/80	20/30	Acrylic	Decentration	27
				180LE		20/80	20/40	Acrylic	-	
14	9	M	Marfan's	160 RE	BL	20/80	20/30	PMMA	PCO	24
				170 LE		20/200	20/30	PMMA	PCO	
15	7	M	Marfan's	210 RE	BL	20/200	20/40	Acrylic	Capsular bag with IOL dislocated	33
				160LE		20/200	20/40	Acrylic	PCO	

* BCVA=Best corrected visual acuity

† UL=Unilateral

‡ BL=Bilateral

§ PCO=Posterior capsule opacification, HM= Hand movement, CF= Counting finger, IOL= Intraocular lens

Problems of phacoaspiration in subluxated crystalline or cataractous lenses are extension of dialysis, vitreous loss, and cortical matter aspiration from capsular bag, inability to implant posterior chamber IOL. Many of the recent studies on phacoemulsification with CTR have shown encouraging results in the management of zonular dialysis in adult patients.[5,15,16] Implantation of CTR stabilizes the capsular bag and allows the surgeon to complete phacoemulsification and IOL implantation.[5,15,16] In various studies, phacoemulsification with CTR implantation was done in patients with subluxation ranging from 90° to over 200°.[5,15,16] In the present study, we had patients with subluxation of 90° to 210°.

Key points to successful CTR implantation are to use high-viscosity visco-elastic material, making the incision at a meridian with intact zonules, to avoid damaging zonular fibers with the movement of the phacotip, perform slow-motion phacoemulsification with a low flow rate, low vacuum, and low bottle height. In our study, high-viscosity visco-elastic sodium hyaluronate 1.4% (Healon GV) was used in all cases and incision was made in the area of intact zonules.

Some surgeons implant CTR after cortical aspiration and before IOL implantation[16] and others prefer to implant CTR before phacoemulsification.[6] In our study, CTR was implanted just after the hydrodissection in all eyes. In an experimental study on cadaveric eyes Ahmed *et al*,[17] have shown that the ideal timing for CTR placement is after lens extraction. Jacob *et al*,[15] found intraoperative extension of the dialysis in 9.52% eyes and in one eye procedure was converted to a lensectomy with three-port pars plana vitrectomy for removal of dropped nuclear fragment. In our study, extension of dialysis occurred in two eyes. Another complication involved during CTR implantation is tear of capsulorrhexis margin. In a study by Praveen *et al*,[16] the authors aborted CTR implantation in two eyes due to anterior capsular tear. In the present study, we did not encounter a similar problem during CTR implantation.

Gimbel *et al*,[6] managed 14 eyes with subluxated cataractous lens with phacoemulsification using CTR. The average age of the patients was 66 years, with a range of 45 to 88 years. Traumatic as well as spontaneous subluxation was included in this study. IOL was centered in all the 14 eyes after a follow-up period ranging from two to 11 months. Cionni *et al*,[5] also showed a similar result. In our study, one eye had decentration of IOL in the immediate postoperative period requiring second procedure to fix one haptic to the sclera. IOL remained well centered in all eyes except one eye which showed increasing decentration of IOL.

In the present study, 16 out of 18 eyes (89%) had a final visual acuity of 20/40 or better. One eye with traumatic cataract had postoperative BCVA of 20/400 because of macular scar and in another eye with idopathic subluxation, visual acuity did not improve beyond 20/60 because of amblyopia. Recently, Georgopoulos *et al*,[18] reported the long-term effect of CTR insertion in adult patients with large traumatic zonular dialysis after phacoemulsification with PCIOL implantation. In their series of 17 eyes of 17 patients, zonular dialysis ranged from 80° to 160°. They did not encounter any intraoperative complication. After a mean follow-up of 25.9 months (range 15 to 35 months) no IOL was found to be decentered, apart from one eye in which the PCIOL was dislocated due to postoperative trauma. Two eyes had poor postoperative visual acuity because of macular pathology.

Konradsen *et al*,[19] managed 37 eyes with ectopia lentis of non-traumatic etiology in children with a Cionni-modified CTR (33 eyes) and conventional CTR (four eyes) and IOL with good long-term results. In their series, 26 eyes needed secondary surgery for visual axis opacification. Two (5.4%) of their eyes needed secondary suturing for IOL dislocation. Vasavada *et al*,[20] reported intraoperative and postoperative performance of endocapsular ring implantation in pediatric eyes with subluxated lens. They included both spontaneous and traumatic subluxatation ranging from 180° to 250°. In all these cases they implanted Cionni CTR and single-piece AcrySof IOL in the capsular bag with good intraoperative performance. Visually significant PCO had developed in 19 eyes (54.3%). Postoperative IOL decentration was observed in three eyes (8.5%) at a mean interval of 28+6.92 months. In our series, nine eyes had visually significant PCO. In six eyes implanted IOL material was PMMA with round-edged optic and three eyes had a square-edged acrylic IOL. In all eyes with PCO, Nd:YAG laser posterior capsulotomy was done to regain optimal visual outcome. CTR may not offer significant prevention of PCO in young children implanted with round-edged or square-edged IOLs. Increasing subluxation and subsequent dislocation of capsular bag with implanted IOL is a challenging situation which ultimately requires removal and vitrectomy and trans-scleral fixation of PCIOL. We had an eye with 210° subluxation, implanted with acrylic hydrophilic IOL and CTR which dislocated with capsular bag after two years, probably due to increasing zonular dehiscence and capsular bag fibrosis. Pars plana vitrectomy with scleral fixation of IOL was done in this case. It is technically difficult to remove CTR from the vitreous cavity as it may damage the retina. Ma *et al*,[21] described two techniques to remove the CTR from the vitreous cavity. In their first case CTR was removed after cutting it into two pieces and in the second case CTR was removed in toto using CTR injector. Lang *et al,*[22] reported removal of posteriorly dislocated CTR intact through sclerotomy site. Surgical approach for dislocated capsular bag-IOL-ring complex depends on CTR position. If the capsular bag-IOL-ring complex is subluxated in the retropulpillary area, CTR-IOL complex can be sutured with sclera through anterior approach and when capsular bag-IOL-ring complex is dislocated completely in the vitreous cavity, pars plana vitrectomy, levitation of CTR-IOL complex with 20-gauge microforceps can be done.[23]

In conclusion, advancement in the technique of phacoaspiraton along with CTR implantation makes capsular bag IOL fixation possible in most of the eyes with subluxated crystalline or cataractous lenses in children. Use of CTR also helps in maintaining IOL centration, stabilization of capsular bag and good postoperative visual recovery. Thus phacoaspiraton with the help of CTR is a safe and predictable procedure in subluxated lens in children. However, elimination of PCO still remains a great challenge in children even after successful phacoaspiraton with CTR implantation. One important concern with implantation of CTR in subluxated lens is long-term follow-up to observe any change in the stability of capsular bag-zonular complex along with CTR and IOL for any chances of increase in dehiscence or dislocation into the vitreous cavity.
